# Fast T Wave Detection Calibrated by Clinical Knowledge with Annotation of P and T Waves

**DOI:** 10.3390/s150717693

**Published:** 2015-07-21

**Authors:** Mohamed Elgendi, Bjoern Eskofier, Derek Abbott

**Affiliations:** 1 Electrical and Computer Engineering in Medicine Group, University of British Columbia and BC Children's Hospital, Vancouver, BC V6H 3N1, Canada; 2 Department of Computing Science, University of Alberta, Edmonton, AB T6G 2E8, Canada; 3 Pattern Recognition Lab, Friedrich-Alexander University Erlangen-Nuernbeg, Haberstr. 2, 91058 Erlangen, Germany; E-Mail: eskofier@cs.fau.de; 4 School of Electrical and Electronic Engineering, University of Adelaide, Adelaide SA 5005, Australia; E-Mail: derek.abbott@adelaide.edu.au

**Keywords:** arrhythmia, affordable healthcare, moving averages

## Abstract

**Background:**

There are limited studies on the automatic detection of T waves in arrhythmic electrocardiogram (ECG) signals. This is perhaps because there is no available arrhythmia dataset with annotated T waves. There is a growing need to develop numerically-efficient algorithms that can accommodate the new trend of battery-driven ECG devices. Moreover, there is also a need to analyze long-term recorded signals in a reliable and time-efficient manner, therefore improving the diagnostic ability of mobile devices and point-of-care technologies.

**Methods:**

Here, the T wave annotation of the well-known MIT-BIH arrhythmia database is discussed and provided. Moreover, a simple fast method for detecting T waves is introduced. A typical T wave detection method has been reduced to a basic approach consisting of two moving averages and dynamic thresholds. The dynamic thresholds were calibrated using four clinically known types of sinus node response to atrial premature depolarization (compensation, reset, interpolation, and reentry).

**Results:**

The determination of T wave peaks is performed and the proposed algorithm is evaluated on two well-known databases, the QT and MIT-BIH Arrhythmia databases. The detector obtained a sensitivity of 97.14% and a positive predictivity of 99.29% over the first lead of the validation databases (total of 221,186 beats).

**Conclusions:**

We present a simple yet very reliable T wave detection algorithm that can be potentially implemented on mobile battery-driven devices. In contrast to complex methods, it can be easily implemented in a digital filter design.

## 1. Introduction

According to the World Health Organization, cardiovascular diseases (CVDs) are the number one cause of death globally; more people die annually from CVDs than from any other cause [1]. An estimated 17.3 million people died from CVDs in 2008, representing 30% of all global deaths [1]. Of these deaths, an estimated 7.3 million were due to coronary heart disease and 6.2 million were due to stroke [1]. Thus, medical researchers have placed significant importance on cardiac health research. This has led to a strong focus on technological advances with respect to cardiac function assessment. One such research pathway is the improvement of conventional cardiovascular diagnosis technologies used in hospitals and clinics.

The most common clinical cardiac test is electrocardiogram (ECG) analysis as it is simple, risk-free, and inexpensive [2]. The signal of each heart beat contains five main waves: the P, Q, R, S, and T waves. The automatic detection of these waves is critical for reliable cardiovascular assessment, such as diagnosing cardiac arrhythmias [3–6], understanding autonomic regulation of the cardiovascular system during sleep and hypertension [7,8], detecting breathing disorders such as obstructive sleep apnea syndrome [9,10], and monitoring other structural or functional cardiac disorders.

The detection of R peaks and QRS complexes has been extensively investigated over the past two decades [11]. Conversely, T wave detection has not been investigated as widely as QRS detection, and the T wave detection problem is still far from being solved [12–15]. Reliable T wave detection is more challenging than QRS complex detection for several reasons, including low amplitudes, low signal-to-noise ratio (SNR), amplitude and morphology variability, and possible overlapping of the P wave and T wave [11,16]. Nevertheless, accurate T wave detection is mandatory for a variety of (differential) diagnostic tasks, such as acute coronary syndrome [17], acute myocardial infarction [18], or potentially fatal arrhythmias [19]. To our knowledge, there was no attempt to develop an ECG detector based on existing clinical knowledge. Thus, in this paper, we investigated the possibility of applying clinical knowledge to build a T wave detection algorithm.

In addition, in the near future, it is expected that Holter devices, which are traditionally used for ECG analysis in the clinic, will be replaced by portable battery-operated devices, such as mobile phones [11]. Therefore, there is a need for a simple, fast, and computationally efficient algorithm to detect arrhythmias efficiently in real time. To develop fast robust algorithms for detecting Arrhythmia in ECG collected by portable, wearable, and battery-driven devices, first we require fully annotated arrhythmia ECG signals as a benchmark for evaluation. Unfortunately, the MIT-BIH Arrhythmia database [20] includes *only* the annotations of R peaks. Therefore, in this study, we annotated T waves in the MIT-BIH Arrhythmia database [20,21]. Moreover, a new fast robust algorithm consisting of two moving averages that are calibrated by a clinical knowledge base is presented.

## 2. Materials and Methods

### 2.1. Data Used

Several standard ECG databases are available for the evaluation of QRS detection algorithms for ECG signals. Most of these databases contain annotated files for R peaks but not for T waves. To demonstrate the applicability of the algorithm presented in this paper, two databases are used in this study: one self-annotated database and one standard annotated database.

#### 2.1.1. Database Annotated Under This Study

An expert manually annotated the P and T peaks of the MIT-BIH Arrhythmia database [20,21] to be used in evaluation for the following reasons:

1. The MIT-BIH database contains 30-min recordings for each patient, which is considerably longer than the records in many other databases, such as the Common Standards for Electrocardiography database, which contains 10-s recordings [22].
2. The MIT-BIH Arrhythmia database contains records of normal ECG signals and records of ECG signals that are affected by non-stationary effects, low SNR, premature atrial complexes, premature ventricular complexes, left bundle blocks, and right bundle blocks. This provides an opportunity to test the robustness of T wave detection methods.
3. The database contains 23 records (the “100 series”) that were chosen at random from a set of more than 4000 24-h Holter tapes, and 25 records (the “200 series”) that were selected from the same set, including a variety of rare and clinically important ECG segments [20]. Several records in the 200 series have abnormal rhythms and QRS morphologies and they suffer from a low SNR. These issues are expected to present significant difficulties for any ECG signal analysis algorithm [20].

**Figure 1:**
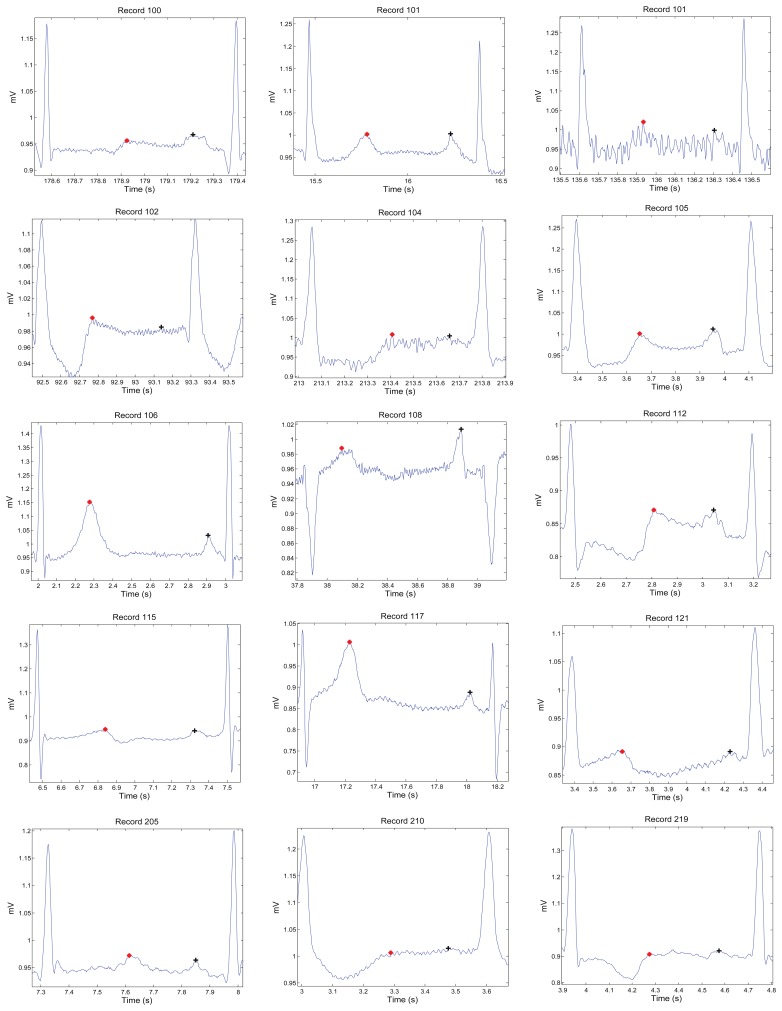
Annotation of P and T waves in normal beats. Here, “+” represents the P wave and “*” represents the T wave. Note that the higher peaks represent the QRS complex.

**Figure 2:**
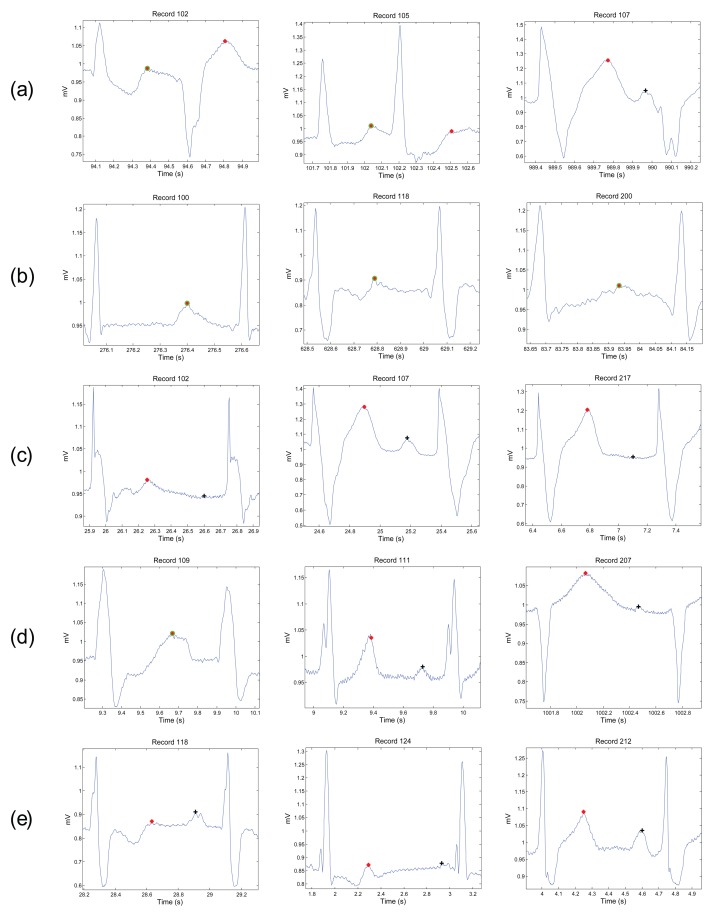
Annotation of Pand T waves in irregular heart beats. Each row contains three different morphologies for a certain type of arrhythmia: (**a**) premature ventricular beats; (**b**) premature atrial beats; (**c**) paced beats; (**d**) left bundle branch block beats; (**e**) right bundle branch block beats. Here, “+” represents the P wave and “*” represents the T wave, while the green circle with asterisk represents merged P and T waves.

**Figure 3:**
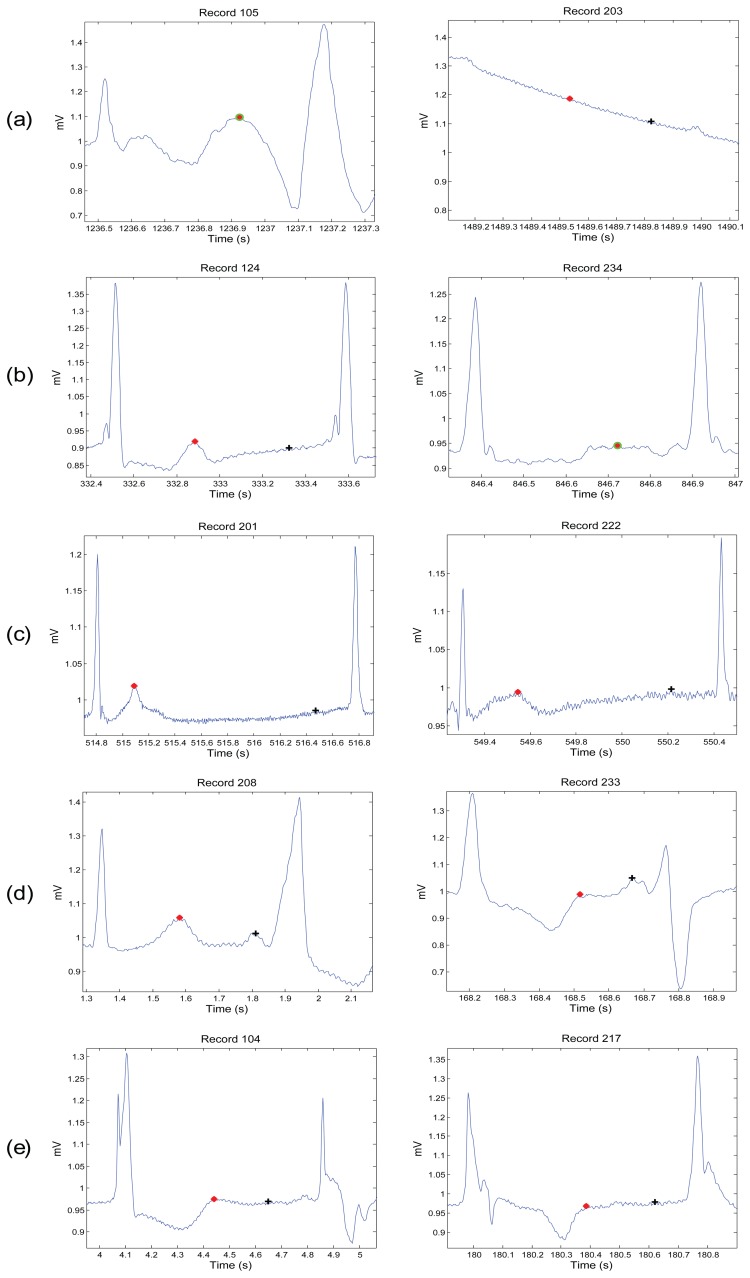
Annotation of P and T waves in unusual beats. Each row contains two different morphologies for a certain type of unusual beats: (**a**) unclassified beats; (**b**) nodal premature beat; (**c**) nodal escape beat; (**d**) fusion of ventricular and normal beat; (**e**) fusion of paced and normal beat. Here, “+” represents the P wave and “*” represents the T wave, while the green circle with asterisk represents merged P and T waves.

Figures 1, 2 and 3 demonstrate examples for annotation of T waves for different beats in the MIT-BIH Arrhythmia database. There was no automated aid provided during the annotation process and only channel one (Lead I) was annotated. For special cases, such as biphasic T waves, the middle point of the wave was considered as a T wave. The annotation file of P and T waves can be downloaded from [23].

#### 2.1.2. Standard Annotated Database

As the MIT-BIH database is self-annotated, the validation of the detector must be carried out using a standard annotated database. For this purpose, the easily-available QT database [24] is used. This database was annotated by two cardiologists and includes different morphologies such as ST change, supraventricular arrhythmia, normal sinus rhythm, sudden death, and long-term ECG signals. The two cardiologists annotated only selected beats (3542 beats in a file called “.q1c”) in all recordings except two recordings: “sel35” and “sel37”. However, the automatic annotation of the whole database was carried using *ecgpuwave* software, which is saved in the “.pu” file. In this work, the T peaks of Lead I of the whole QT database are used for validation as they are more salient and certain compared to the onset and offset. In this work, the T peaks of the whole QT database are used for validation as they are more salient and certain compared to the onset and offset. Moreover, once the T peak is detected correctly, searching for the onset and offset is a relatively easy step; however, this is not the focus of this study.

### 2.2. T Wave Detection Algorithm

**Figure 4:**
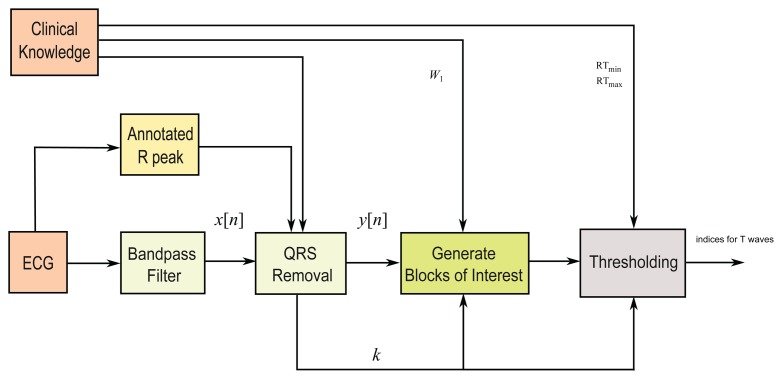
Structure of the T waves detection algorithm. The algorithm consists of three main stages: pre-processing (clinical knowledge, bandpass filtering, squaring, and QRS removal), feature extraction (generating potential blocks using two moving averages), and classification (thresholding).

In this study, a fast robust knowledge-based T wave detection algorithm is discussed and evaluated. The algorithm is based on the framework proposed by Elgendi for detecting QRS complexes in ECG signals [25,26], for detecting systolic waves in photoplethysmogram signals [27], detecting *a* waves [28], and detecting *c*, *d*, *e* waves [29] in acceleration photoplethysmogram (PPG) signals. We build upon this approach to detect T waves. The method consists of three main stages: pre-processing (clinical knowledge, bandpass filtering, squaring, and QRS removal), feature extraction (generating potential blocks using two moving averages), and classification (thresholding). The structure of the algorithm is given in Figure 4.

#### 2.2.1. Bandpass Filter

Most of the energy of T waves lies below 10 Hz [30,31]; thus, a zero-phase second-order Butterworth filter, with a 0.5–10 Hz bandpass, is implemented to remove the baseline wander and high frequencies that do not contribute to the T waves. The output of the zero-phase Butterworth filter applied to the ECG signal produces a filtered signal *x*[*n*].

#### 2.2.2. QRS Removal

The QRS removal step based on the relative distance of the T wave to its associated R peak. Removing the QRS complex has two advantages: (1) T waves become the dominant feature in the processed signal; and (2) it simplifies the search for T waves relative to the position of R peaks. In this study, as proof of concept, the R peaks provided in the MIT-BIH Arrhythmia and QT databases are used. Removing the QRS complex is performed by setting the signal to zero for the duration of the QRS complex.

**Figure 5:**
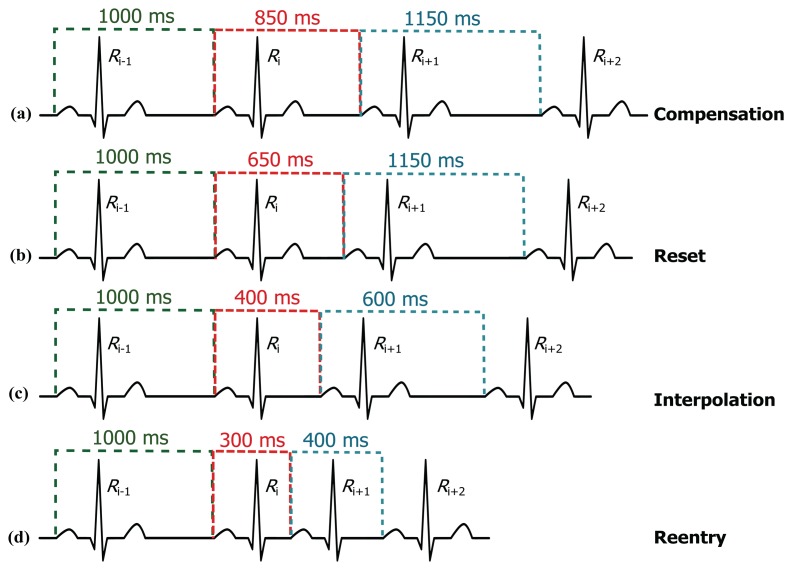
Types of sinus node response to atrial premature depolarization (adapted from [32]).

The limits (thresholds) of the RT distance are determined using ECG clinical knowledge. The signal *y*[*n*] is initialized as equal to the filtered ECG *x*[*n*] signal. The QRS removal length thresholds are determined based on the clinical phases of the RR intervals. As the duration of the QRS complex varies with the heart beat type, a clinical database is required to remove the QRS complex, according to its type. Roskamm and Csapo divided the ECG into four phases: compensation, reset, interpolation, and reentry [32]. Based on their analysis, in the compensation phase (Figure 5a), the second beat (850 ms) is followed by a prolonged beat (1150 ms) to compensate the two beats duration (2000 ms). During the reset phase (Figure 5b), the second beat (650 ms) is followed by a prolonged beat (1150 ms), while in the interpolation (Figure 5c), the second beat (400 ms) is followed by an irregular beat (600 ms). Finally, in the reentry phase (Figure 5d), the second beat (300 ms) is followed by a rapid irregular beat (400 ms); however, an extra category is added to capture complex arrhythmias (repetitive, bigeminy, or trigeminy). The output of this stage will produce an updated *y*[*n*] signal.

**Figure 6:**
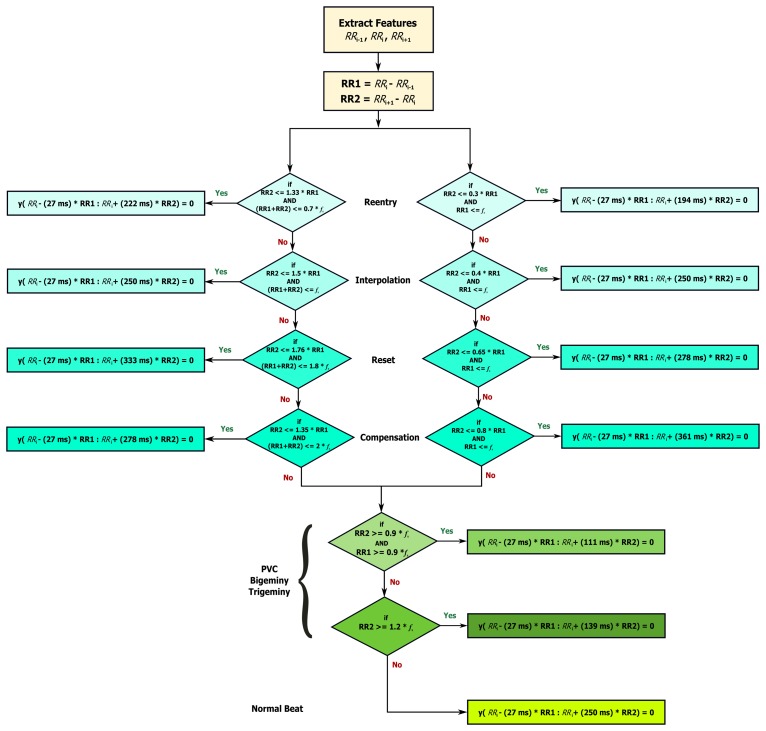
Rule-based knowledge representation of QRS removal based on the clinical knowledge shown in Figure 5.

Based on the clinical information presented in Figure 5, the ratio of the second beat duration to the first and third beat duration generates a rule-based knowledge representation, as shown in Figure 6. For example, in the case of reentry (left branch of the flowchart in Figure 6), the first threshold is calculated by dividing the duration between the third and the fourth beat (*R_i_*_+2_ − *R_i_*_+1_ = 400 ms) by the duration between the two and the third beat (*R_i_*_+1_ − *R_i_* = 300 ms) resulting as 1.33; however, this value was calculated with reference to the duration between first beat and second beat (*R_i_* − *R_i_*_−1_ = 1000 ms)—this generates the first rule: RR2 ≤ 1.33 RR1, where RR1 = *R_i_* − *R_i_*_−1_, RR2 = *R_i_*_+1_ − *R_i_*, and *i* is the beat index. The second threshold is the expected total duration of the second and third beats (300 ms + 400 ms), which equals 0.7 s—this generates the second rule: (RR1 + RR2) ≤ 0.7*f_s_*. On the right side the flowchart in Figure 6, in the case of reentry, the first threshold is calculated by dividing the duration between the third and the fourth beat (*R_i_*_+1_ − *R_i_* = 300 ms) by the duration between the first and the second beat (*R_i_* − *R_i_*_−1_ = 1000 ms) resulting as 0.3—this generates the first rule: RR2 ≤ 0.3 RR1, while the second threshold is based on the duration between the first and the second beat (*R_i_* − *R_i_*_−1_ = 1000 ms) which is 1 s (or *f_s_*)—this generates the second rule: RR1 ≤ *f_s_*. Similarly, all other rules were created for the other four ECG phases.

During the QRS removal, the RR interval that satisfied each category is saved and referred to as RR*_k_*, where *k* is the category type (compensation, reset, interpolation, reentry, and complex arrhythmias). The normalized RR intervals average in each category is calculated as $M_{c}=\left(\sum_{j=1}^{l}{\text{RR}}_{c,j}\right)/\left(lf_{s}\right)$, where *l* is the number of RR intervals saved in category *c*, and *f_s_* is the sampling frequency Dividing *M_c_* by *f_s_* is sufficient as it is equivalent to the average RR interval in healthy subjects.

#### 2.2.3. Generating Blocks of Interest

**Figure 7:**
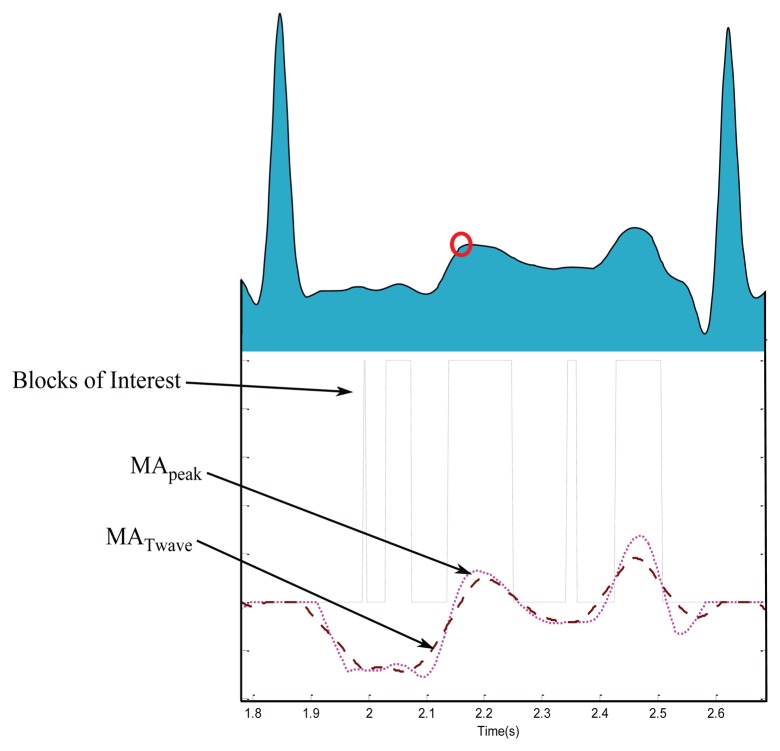
Demonstrating the effectiveness of using two moving averages to detect T waves. The dotted line is the first moving average, while the dashed line is the second moving average. The red circle is the detected T wave peak.

Blocks of interest are generated using two event-related moving averages that demarcate the areas of T waves, a method which was first introduced in [33]. The particular method used to generate blocks of interest has been mathematically shown to detect *a* waves [28], QRS complexes [25], and systolic waves in PPG signals [27]. In this procedure, the first moving average (MA_peak_) is used to emphasize the peak of the T wave area, as the dotted signal shown in Figure 7, and is given by

(1) $${\text{MA}}_{\text{peak}}\left[n\right]=\frac{1}{W_{1}}\left(y\left[n-\left(W_{1}-1\right)/2\right]+\cdots +y\left[n\right]+\cdots +y\left[n+\left(W_{1}-1\right)/2\right]\right)$$

where *W*_1_ represents the window size of approximately the peak duration of the T wave in ECG signals. The initial value for *W*_1_ of 70 ms is determined by Trahanias [34]. However, as the ECG signals may contain different arrhythmias the value of *W*_1_ will be calculated relative to the most frequent RR intervals in all five categories ( $k=\underset{c}{\max}M_{c}$). Then, the value of *W*_1_ = (70 ms × *f_s_*) × *k*, and the result is rounded to the nearest odd integer. The second moving average (MA_Twave_) is used to emphasize the T wave area to be used as a threshold for the first moving average, shown as a dashed signal Figure 7, and is given by

(2) $${\text{MA}}_{\text{Twave}}\left[n\right]=\frac{1}{W_{2}}\left(y\left[n-\left(W_{2}-1\right)/2\right]+\cdots +y\left[n\right]+\cdots +y\left[n+\left(W_{2}-1\right)/2\right]\right)$$

where *W*_2_ represents the window size of approximately the T wave duration. The initial value for *W*_2_ of 140 ms is determined by Laguna *et al.* [35]. However, as the ECG signals may contain different arrhythmias, the value of *W*_2_ will be calculated relative to the most frequent RR intervals in all five categories (*k*). Then, the value of *W*_2_ = (140 ms × *f_s_*) × *k*, and the result is rounded to the nearest odd integer. For example, the total values of *W*_1_ and *W*_2_ for detecting T waves in record 100 from MIT-BIH Arrhythmia database were 20 samples (55.6 ms) and 40 samples (111.2 ms); respectively.

#### 2.2.4. Thresholding

In this stage, the blocks of interest are generated by comparing the MA_peak_ signal with MA_Twave_. Many blocks of interest will be generated, some of which will contain the T wave and others will contain P waves, U waves, and noise. Therefore, the next step is to reject blocks that result from noise. Rejection is based on the relative positions of P and T waves to R peaks and anticipated peak width.

**Figure 8:**
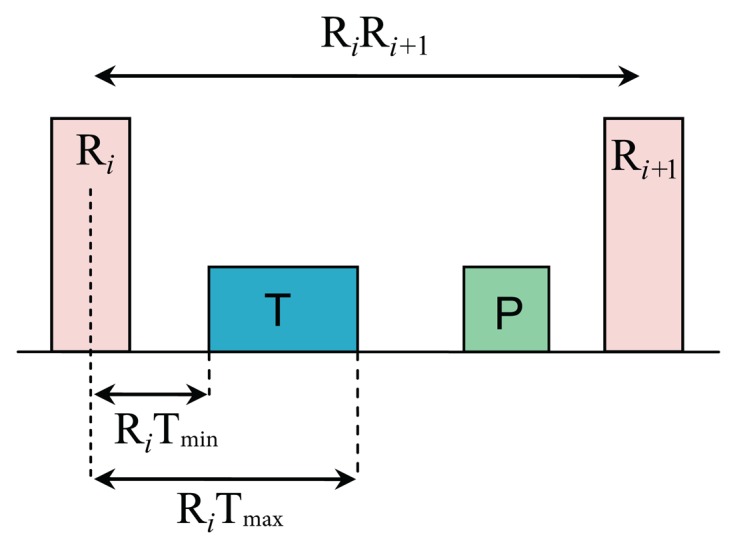
Search regions for T waves in terms of time occurrence with respect to the current R peak (R*_i_*) and the next R peak (R*_i_*_+1_). Where R*_i_*T_min_ represents the minimum interval between the T wave and current R peak and R*_i_*T_max_ represents the maximum interval between the T wave and the current R peak.

To determine whether the detected blocks contain T waves or not, the number of blocks in each consecutive RR interval is counted. A threshold based on the distance of the maximum point within a block to the R peak is applied to distinguish P waves from T waves and noise, as shown in Figure 8. The search regions for T waves in terms of time occurrence with respect to the current R peak (R*_i_*) and the next R peak (R*_i_*_+1_) are calculated as

(3) $${\text{R}}_{i}{\text{T}}_{\min}={\text{D}}_{\min}{\text{R}}_{i}{\text{R}}_{i+1}$$

(4) $${\text{R}}_{i}{\text{T}}_{\max}={\text{D}}_{\max}{\text{R}}_{i}{\text{R}}_{i+1}$$

where R*_i_*T_min_ represents the minimum dynamic interval between the T wave and the current R peak, R*_i_*T_max_ represents the maximum dynamic interval between the T wave and the current R peak, while R*_i_*R*_i_*_+1_ represents the interval between *R_i_* and *R_i_*_+1_. The exact values for D_min_ and D_max_ are 170 ms and 800 ms, respectively, as determined by Schimpf *et al.* [36] to represent the minimum RT durations for subjects with arrhythmia and maximum RT duration for healthy subjects. All detected blocks go through a durational threshold to reject the undesired blocks called THR_1_, which rejects the blocks that contain P wave, U wave, and noise. By applying the THR_1_ threshold, the accepted blocks will contain T peaks only,

(5) $${\text{THR}}_{1}=W_{1}$$

**Figure 9:**
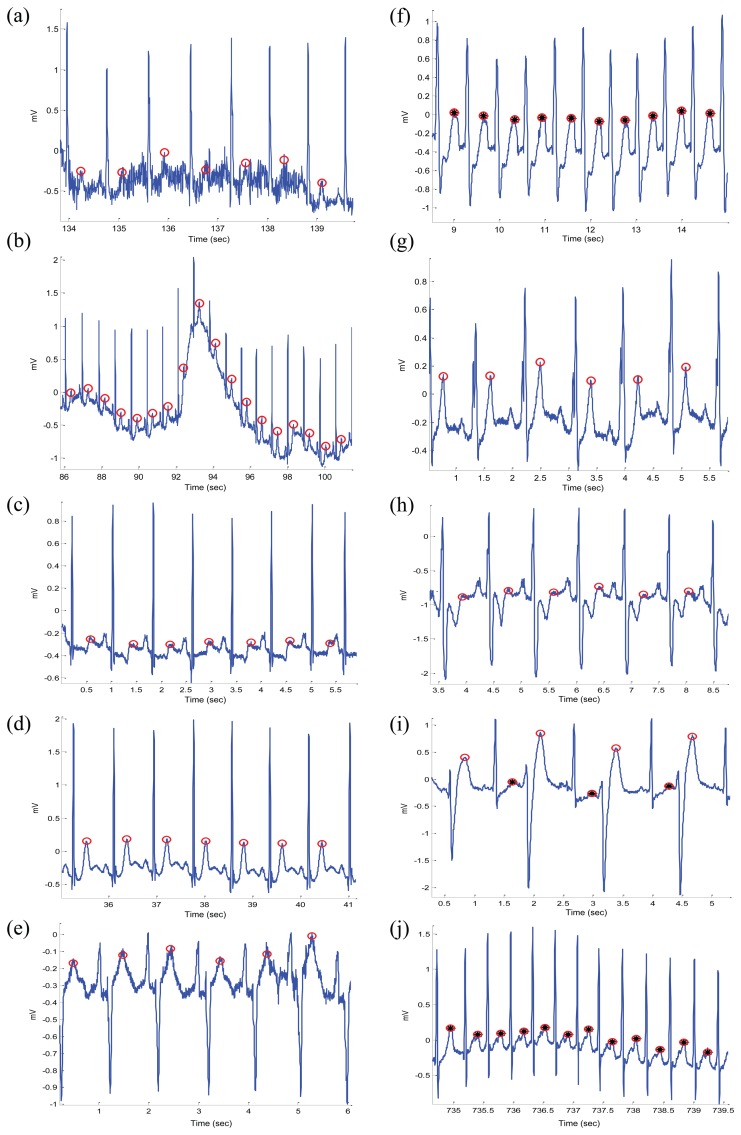
Demonstrating the performance of the proposed T wave detection algorithm on the MIT-BIH Arrhythmia database. The algorithm succeeds to detect T wave peaks in electrocardiogram (ECG) signals that contain: (**a**) high-frequency noise; (**b**) baseline wander; (**c**) normal sinus rhythm without U waves; (**d**) normal sinus rhythm with U waves; (**e**) normal sinus rhythm with negative polarization; (**f**) left bundle branch block (LBBB) beats with merged P and T waves; (**g**) LBBB beats; (**h**) right bundle branch block (RBBB) beats from record 118; (**i**) premature ventricular contraction (PVC) beats from record 200; (**j**) premature atrial contraction (PAC) beats from record 209. Here, the empty red circle represents the detected T wave while the a circle with a black asterisk represents detection of merged P and T waves.

After applying the relative-position thresholds, there are three possibilities for the number of detected blocks within the area of interest:

1. **Zero:** if there is no block detected, it means the algorithm failed to detect a T wave in the current RR interval.
2. **One:** if there is one detected block, it means the algorithm succeeds in detecting T wave, P and T waves are most likely merged within one block, which is marked as a circle with a black asterisk inside (see Figure 9i,j).
3. **More than one:** if there are multiple detected blocks, as shown in Figure 7, it means one of the detected blocks contains T waves. However, in this work the nearest block to the current R peak is considered a T wave.

The last stage is to find the maximum absolute value within each block to detect the peak of T wave. The detected T wave peaks are compared to the annotated T wave peaks to determine whether they were detected correctly. The search range for the true T wave peak is fixed to ±30 ms for both databases, to ensure consistency of comparison. The search region of 30 ms is good enough for diagnostics as it is less than *W*_1_.

## 3. Results

The algorithm was evaluated using the MIT-BIH database. The T waves were detected successfully even when the P and T waves are merged in Arrhythmia ECG signals that are affected by: high-frequency noise, baseline wander, normal sinus rhythm (NSR), left bundle branch block (LBBB), right bundle branch block (RBBB), premature ventricular contraction (PVC), and premature atrial contraction (PAC). All of the reasons for detection failure are described below. High-frequency noise results from the instrumentation amplifiers, recording system, and ambient electromagnetic signals received by the cables. The signal shown in Figure 9a has been corrupted by power-line interference at 60 Hz and its harmonics and other high frequencies. It can be seen that the proposed algorithm is robust to noise. Moreover, the proposed algorithm is not sensitive to baseline wander and detected the T waves correctly, as shown in Figure 9b. This is because the moving averages were applied to the bandpass filtered ECG signal—which is discussed in the Bandpass Filter Subsection.

The NSR is a normal ECG cycle; it is initiated by the sinoatrial node and consists of a P wave followed, after a brief pause, by a QRS complex and then a T wave [37]. The proposed algorithm correctly detected T waves in three types of normal beats: (1) NSR without U waves (record 100 of the MIT-BIH database), as shown in Figure 9c; (2) NSR with U waves (record 103), as shown Figure 9d; and (3) NSR with negative polarization (record 108), as shown Figure 9e. The LBBB results from conduction delays or blocks at any site in the intraventricular conduction system, including the main LBBB and the bundle of His. The result of an LBBB is an extensive reorganization of the activation pattern of the left ventricles [37]. The proposed algorithms successfully detected normal and merged P and T waves in two types of LBBBs: (1) LBBB beats with merged P and T waves (record 109), as shown in Figure 9f and (2) LBBB beats with normal T waves (record 111), as shown in Figure 9g. However, RBBB is a result of a conduction delay in a portion of the right-sided intra-ventricular conduction system. The delay can occur in the main RBBB itself, in the bundle of His, or in the distal right ventricular conduction system. The RBBBs may be caused by a minor trauma, such as right ventricular catheterization [37]. As shown in Figure 9h, the proposed algorithms succeeded in detecting the T waves in ECG signals of RBBB (record 118).

The PVCs are characterized by the premature occurrence of a QRS complex that is abnormal in shape, and that has a longer duration than normal QRS complexes, generally exceeding 120 ms [37]. The T wave is commonly large and opposite in direction to the major deflection of the QRS. In general, the QRS complex is not preceded by a P wave, but it can be preceded by a non-conducted sinus P wave occurring at the expected time [37]. In Figure 9i, a special case of PVC is shown, called bigeminy, where the premature ventricular beats occur after every normal beat in an alternating pattern.

The proposed algorithm succeeded in detecting the T waves in the normal beats and the T waves in the premature ventricular beats (record 200). Note that PACs are among the most common causes of irregular pulses and can originate from any area in the heart [37]. The impulse is discharged prematurely by an irritable focus in the atria giving rise to a distorted P wave, usually superimposed on the preceding T wave. As shown in Figure 9j, the proposed algorithms detected the merged T waves in PACs (record 209). As illustrated in Figure 9, the proposed method successfully detected T waves in ECG signals with a low SNR, baseline wander, and various arrhythmias. The performance of the T wave detection algorithms is evaluated using two statistical measures: SE = TP/(TP + FN) and +P = TP/(TP + FP), where TP is the number of true positives (T wave peak detected within the range of 30 ms of the annotated T wave peak), FN is the number of false negatives (annotated T wave peak has not been detected), and FP is the number of false positives (T wave peak detected outside the range of 30 ms of the annotated T wave peak). The sensitivity SE reports the percentage of true beats that were correctly detected by the algorithm. The positive predictivity +P reports the percentage of beat detections that were true beats.

The abnormal heart rhythms caused a large number of FNs compared to the FPs. Table 1 shows the result of T wave detection over 48 records of the MIT-BIH database. FNs are mainly caused by noise and PVC, as in record 219, and atrial fibrillation, as in record 202. The algorithm achieved a sensitivity of 99.86% and a positive predictivity of 99.65%, which are promising results for handling the non-stationary effects, low SNR, PACs, PVCs, LBBBs, and RBBBs in ECG signals.

### 3.1. Comparison of Performance on the QT Database

The detection performance on the QT database obtained by the proposed T wave detector record by record performance is shown in Tables 2 and 3. The overall comparison of our results with the existing T wave detection algorithms on the QT database is demonstrated in Table 4. It summarizes the performances in terms of number of beats, methodology, SE, and +P. Note that the proposed algorithm scored slightly higher overall performances (average of SE and +P) than Martínez *et al.* [38] and Laguna *et al.* [39] over the manually annotated T waves. It is clear that the proposed algorithm succeeds in handling long ECG recordings with high performance over the 111,201 automatically annotated heart beats. Moreover, the proposed T wave detector has not been re-tuned over any databases, thus the results are promising, and the algorithm can detect T peaks over different databases, sampling frequencies, types of arrhythmias, and noise.

### 3.2. Processing Time

Less computational time is achieved when the simplest method is used (*i.e.*, the algorithm presented in this paper requires less computational time). This is advantageous in terms of future development of wearable and portable diagnostic devices, and in terms of helping online and real-time diagnoses.

It is misleading to suggest that mentioning the average speed of the proposed detector, over a certain time length of ECG signal, would provide a comparative result. This is because the processing time depends on the number of beats within each ECG recording, not on the record length. In this study, the T wave detector was implemented in MATLAB 2010b (The MathWorks, Inc., Natick, MA, USA) on Intel™ i5 CPU 2.27 GHz.

It is worth noting that the number of beats of the 15-min recordings category in QT database was relatively consistent—with a mean ± SD, number of beats 1059 ± 275—over all records of this category. On the contrary, the 30-min beat average, in MIT-BIH database, was 2291 with an SD of 448 beats. As the processing time depends on the number of beats rather than the recording length [25], we found, for example, the proposed detector took 2.58 s to process record 215-MITDB contains 3362 beats, while it took 0.28 s to process 527 beats in record 33-QTDB. In general, without taking the number of beats into consideration, the speed of the proposed detector is fast. The suggested detector handles 15-min recordings in 0.52 ± 0.14 s, while it takes 1.98 ± 0.32 s to handle 30-min ECG recordings.

## 4. Limitations of Study and Future Work

The preliminary results are promising, especially after testing the algorithm on the QT database; however, testing the algorithm on a larger sample size is necessary to generalize the findings. In addition, a more focused study is needed to investigate atrial fibrillation, atrial flutter, paroxysmal supraventricular tachycardia, junctional tachycardia, and multifocal atrial tachycardia as the morphology of T waves may differ.

The presented method assumes that the R peaks are correctly detected. There is a linear correlation between the detection of T waves and the detected R peaks. If the R peaks are misclassified, this method will fail as it depends on the position of R peaks. However, this study provides a positive proof of concept for detecting T waves in arrhythmic ECG beats.

We created a rule-based system based on the three RR interval window proposed in [32]. Perhaps, it is important to investigate the development of a rule-based system based on more than three RR intervals to examine if it will improve the overall T wave detection accuracy. Moreover, the proposed method is based on several thresholds, which are calibrated using clinical knowledge. Clinical knowledge is considered the gold standard in our work; thus, the obtained thresholds were considered optimal. However, an open question for future work is to optimize all thresholds to improve accuracy.

One of the next steps regarding the results of this study is to detect arrhythmic ECG beats using the RT or ST interval as a main feature. In addition, the detection of P waves based on the accurate detection of T wave peaks needs to be examined. Moreover, perhaps, an optimization over the clinical parameters after splitting the databases into a training set and test set may improve the detection rate of the T waves. There is also a need to investigate the T-waves with different morphology, e.g., biphasic T-waves.

Technically, exploring the event-related moving average methodology for detecting events in ECG signals is promising in terms of computational complexity and efficiency. This can be further improved by investigating other bandpass filters with different orders and also by developing fast-moving average (or median) techniques for real-time analysis and mobile phone applications.

## 5. Conclusions

There is a limitation when evaluating T wave detection algorithms as datasets with annotated T waves are lacking. Consequently, comparing existing algorithms becomes even more difficult. Therefore, annotation of T waves is discussed and provided. Using these approaches, it is possible to support diagnostic analysis, delivering important information for (differential) diagnosis to medical experts. Using mobile technologies with automatic analysis software driven by medical expert knowledge, it will be furthermore possible to provide screening and monitoring solutions in places where medical expertise is scarce, such as remote rural areas and developing countries.

The use of two moving averages is simple and computationally efficient for mobile electronic health tools, such as cell phones and telemedicine technologies. The assessment of the T detector has been reliably carried out over the existing standard databases (QT and MIT-BIH), which contain different beat types and morphologies found in ECG signals. The developed algorithm was evaluated on all ECG recordings in the MIT-BIH database, 48 self-annotated records containing a total of 109,985 heart beats. It achieved a sensitivity of 95% and a positive predictivity of 98.59% over the MIT-BIH ECG signals, which contain low SNR, baseline wander, paced beats, and various arrhythmias. Interestingly, the proposed algorithm succeeded in scoring the highest overall accuracy of 98.84% over the manually annotated QT database (3542 heart beats) when compared to the other three algorithms (*cf.* Table 4). Moreover, the algorithm scored a high overall accuracy of 96.7% over the automaticlly annotated QT database (111,201 heart beats). Overall, simplicity and efficiency are required in developing T wave detection algorithms for processing long-term recordings and large databases as well as for expanding our telemedicine capabilities in the near future.

## Figures and Tables

**Table 1:** T wave peak detection performance over the annotated MIT-BIH Arrhythmia database [20,21]. To evaluate the performance of the T wave detection algorithm, two statistical measures are used: SE = TP/(TP + FN) and +P = TP/(TP + FP), where TP is the number of true positives (T wave peak detected within the range of 30 ms of the annotated T wave peak), FN is the number of false negatives (annotated T wave peak has not been detected), and FP is the number of false positives (T wave peak detected outside the range of 30 ms of the annotated T wave peak).

**Record**	**No of beats**	**TP**	**FP**	**FN**	**SE (%)**	**+P (%)**
**100**	2274	2272	0	0	100.00	100.00
**101**	1866	1863	1	4	99.79	99.95
**102**	2187	2185	0	0	100.00	100.00
**103**	2084	2082	0	4	99.81	100.00
**104**	2229	2227	0	1	99.96	100.00
**105**	2602	2586	0	2	99.92	100.00
**106**	2026	2024	0	56	97.23	100.00
**107**	2136	2134	0	3	99.86	100.00
**108**	1763	1757	0	13	99.26	100.00
**109**	2533	2530	0	0	100.00	100.00
**111**	2123	2121	0	16	99.25	100.00
**112**	2539	2537	0	0	100.00	100.00
**113**	1794	1792	0	0	100.00	100.00
**114**	1890	1885	0	69	96.34	100.00
**115**	1953	1951	0	19	99.03	100.00
**116**	2395	2392	0	2	99.92	100.00
**117**	1535	1533	0	0	100.00	100.00
**118**	2278	2276	0	4	99.82	100.00
**119**	1988	1986	0	4	99.80	100.00
**121**	1863	1860	0	46	97.53	100.00
**122**	2476	2474	0	0	100.00	100.00
**123**	1519	1517	0	0	100.00	100.00
**124**	1619	1617	0	7	99.57	100.00
**200**	2601	2599	0	9	99.65	100.00
**201**	1949	1947	0	57	97.07	100.00
**202**	2138	2134	0	113	94.70	100.00
**203**	2988	2965	0	1	99.97	100.00
**205**	2656	2556	0	0	100.00	100.00
**207**	2324	2139	0	9	99.58	100.00
**208**	2953	2949	0	0	100.00	100.00
**209**	3006	3003	0	5	99.83	100.00
**210**	2652	2637	0	0	100.00	100.00
**212**	2748	2746	0	0	100.00	100.00
**213**	3250	3247	0	0	100.00	100.00
**214**	2262	2184	0	0	100.00	100.00
**215**	3362	3354	0	0	100.00	100.00
**217**	2208	2205	0	3	99.86	100.00
**219**	2154	2152	0	144	93.31	100.00
**220**	2048	2046	0	2	99.90	100.00
**221**	2427	2424	0	0	100.00	100.00
**222**	2485	2472	0	33	98.67	100.00
**223**	2604	2601	0	1	99.96	100.00
**228**	2060	2056	0	52	97.47	100.00
**230**	2256	2254	0	39	98.27	100.00
**231**	1571	1569	0	0	100.00	100.00
**232**	1783	1781	0	1	99.94	100.00
**233**	3077	2914	0	1	99.97	100.00
**234**	2751	2749	0	0	100.00	100.00

	109985	109284	1	720	99.28	100.00

**Table 2:** T wave peak detection performance over the manually annotated 11 recordings of the QT database [24]. To evaluate the performance of the T wave detection algorithm, two statistical measures are used: SE = TP/(TP + FN) and +P = TP/(TP + FP), where TP is the number of true positives (T wave peak detected within the range of 30 ms of the annotated T wave peak), FN is the number of false negatives (annotated T wave peak has not been detected), and FP is the number of false positives (T wave peak detected outside the range of 30 ms of the annotated T wave peak).

**Record**	**No of beats**	**TP**	**FP**	**FN**	**SE (%)**	**+P (%)**
**sel100**	30	30	0	0	100.00	100.00
**sel102**	85	85	0	0	100.00	100.00
**sel103**	30	30	0	0	100.00	100.00
**sel104**	77	75	0	0	100.00	100.00
**sel114**	50	50	0	0	100.00	100.00
**sel116**	50	49	0	0	100.00	100.00
**sel117**	30	30	0	0	100.00	100.00
**sel123**	30	30	0	0	100.00	100.00
**sel213**	71	71	0	0	100.00	100.00
**sel221**	30	29	1	1	96.67	96.67
**sel223**	31	31	0	0	100.00	100.00
**sel230**	50	42	9	8	84.00	82.35
**sel231**	50	50	0	0	100.00	100.00
**sel232**	30	30	0	0	100.00	100.00
**sel233**	30	30	0	0	100.00	100.00
**sel301**	30	30	0	0	100.00	100.00
**sel302**	30	30	0	0	100.00	100.00
**sel306**	36	32	0	0	100.00	100.00
**sel307**	30	30	0	0	100.00	100.00
**sel308**	50	40	10	10	80.00	80.00
**sel310**	30	30	0	0	100.00	100.00
**sel803**	30	30	4	0	100.00	88.24
**sel808**	30	30	0	0	100.00	100.00
**sel811**	30	30	0	0	100.00	100.00
**sel820**	30	30	0	0	100.00	100.00
**sel821**	30	30	0	0	100.00	100.00
**sel840**	70	70	0	0	100.00	100.00
**sel847**	33	33	0	0	100.00	100.00
**sel853**	30	30	0	0	100.00	100.00
**sel871**	70	70	0	0	100.00	100.00
**sel872**	30	30	0	0	100.00	100.00
**sel873**	33	33	0	0	100.00	100.00
**sel883**	30	30	0	0	100.00	100.00
**sel891**	71	71	0	0	100.00	100.00
**sel16265**	30	30	0	0	100.00	100.00
**sel16272**	30	30	0	0	100.00	100.00
**sel16273**	30	30	0	0	100.00	100.00
**sel16420**	30	30	0	0	100.00	100.00
**sel16483**	30	30	0	0	100.00	100.00
**sel16539**	30	30	0	0	100.00	100.00
**sel16773**	30	21	9	9	70.00	70.00
**sel16786**	30	30	0	0	100.00	100.00
**sel16795**	30	30	0	0	100.00	100.00
**sel17453**	30	30	0	0	100.00	100.00
**sele0104**	30	30	0	0	100.00	100.00
**sele0106**	30	30	0	0	100.00	100.00
**sele0107**	34	34	0	0	100.00	100.00
**sele0110**	30	30	0	0	100.00	100.00
**sele0111**	30	30	0	0	100.00	100.00
**sele0112**	50	50	0	0	100.00	100.00
**sele0114**	30	30	0	0	100.00	100.00
**sele0116**	30	30	0	0	100.00	100.00
**sele0121**	30	30	0	0	100.00	100.00
**sele0122**	30	30	0	0	100.00	100.00
**sele0124**	50	50	0	0	100.00	100.00
**sele0126**	30	25	4	5	83.33	86.21
**sele0129**	30	30	0	0	100.00	100.00
**sele0133**	30	30	0	0	100.00	100.00
**sele0136**	30	30	0	0	100.00	100.00
**sele0166**	36	36	0	0	100.00	100.00
**sele0170**	30	30	0	0	100.00	100.00
**sele0203**	30	30	0	0	100.00	100.00
**sele0210**	30	30	0	0	100.00	100.00
**sele0211**	30	30	0	0	100.00	100.00
**sele0303**	30	30	0	0	100.00	100.00
**sele0405**	30	30	0	0	100.00	100.00
**sele0406**	31	31	0	0	100.00	100.00
**sele0409**	30	30	0	0	100.00	100.00
**sele0411**	30	30	0	0	100.00	100.00
**sele0509**	30	30	0	0	100.00	100.00
**sele0603**	30	30	0	0	100.00	100.00
**sele0604**	30	30	0	0	100.00	100.00
**sele0606**	30	30	0	0	100.00	100.00
**sele0607**	30	30	0	0	100.00	100.00
**sele0609**	30	30	0	0	100.00	100.00
**sele0612**	30	30	0	0	100.00	100.00
**sele0704**	30	30	0	0	100.00	100.00
**sel30**	30	30	0	0	100.00	100.00
**sel31**	30	26	4	4	86.67	86.67
**sel32**	30	30	0	0	100.00	100.00
**sel33**	30	30	0	0	100.00	100.00
**sel34**	30	30	0	0	100.00	100.00
**sel36**	31	30	1	1	96.77	96.77
**sel38**	30	30	0	0	100.00	100.00
**sel40**	30	30	1	0	100.00	96.77
**sel41**	30	30	0	0	100.00	100.00
**sel42**	30	30	0	0	100.00	100.00
**sel43**	30	28	0	0	100.00	100.00
**sel44**	30	30	0	0	100.00	100.00
**sel45**	30	29	1	1	96.67	96.67
**sel46**	30	30	0	0	100.00	100.00
**sel47**	30	27	2	2	93.10	93.10
**sel48**	30	30	0	0	100.00	100.00
**sel49**	30	30	0	0	100.00	100.00
**sel50**	30	30	0	0	100.00	100.00
**sel51**	32	32	0	0	100.00	100.00
**sel51**	30	30	0	0	100.00	100.00
**sel52**	30	30	0	0	100.00	100.00
**sel17152**	30	30	0	0	100.00	100.00
**sel14046**	31	31	0	0	100.00	100.00
**sel14157**	30	30	0	0	100.00	100.00
**sel14172**	50	50	0	0	100.00	100.00
**sel15814**	30	30	0	0	100.00	100.00

	3542	3491	46	41	98.90	98.77

**Table 3:** T wave peak detection performance over the automatically annotated QT database [24]. To evaluate the performance of the T wave detection algorithm, two statistical measures are used: SE = TP/(TP + FN) and +P = TP/(TP + FP), where TP is the number of true positives (T wave peak detected within the range of 30 ms of the annotated T wave peak), FN is the number of false negatives (annotated T wave peak has not been detected), and FP is the number of false positives (T wave peak detected outside the range of 30 ms of the annotated T wave peak).

**Record**	**No of beats**	**TP**	**FP**	**FN**	**SE (%)**	**+P (%)**
**sel100**	1134	1132	0	1	99.91	100.00
**sel102**	1088	1086	0	2	99.82	100.00
**sel103**	1048	1046	4	5	99.52	99.62
**sel104**	1109	1107	9	10	99.10	99.19
**sel114**	867	864	1	7	99.19	99.88
**sel116**	1186	1184	0	25	97.89	100.00
**sel117**	766	764	0	1	99.87	100.00
**sel123**	756	754	0	0	100.00	100.00
**sel213**	1641	1639	1	2	99.88	99.94
**sel221**	1247	1244	0	116	90.68	100.00
**sel223**	1309	1307	0	6	99.54	100.00
**sel230**	1077	1075	115	200	81.41	88.40
**sel231**	732	730	0	1	99.86	100.00
**sel232**	866	864	18	19	97.80	97.92
**sel233**	1532	1265	13	112	91.79	98.97
**sel301**	1352	1348	0	0	100.00	100.00
**sel302**	1501	1498	1	2	99.87	99.93
**sel306**	1040	1038	0	30	97.11	100.00
**sel307**	853	851	0	1	99.88	100.00
**sel308**	1294	1292	19	21	98.38	98.53
**sel310**	2012	2008	0	3	99.85	100.00
**sel803**	1026	1024	0	84	91.80	100.00
**sel808**	903	901	24	29	96.78	97.32
**sel811**	704	702	0	1	99.86	100.00
**sel820**	1159	1157	1	3	99.74	99.91
**sel821**	1557	1555	2	3	99.81	99.87
**sel840**	1180	1178	1	2	99.83	99.92
**sel847**	803	799	0	3	99.62	100.00
**sel853**	1113	1110	6	8	99.28	99.46
**sel871**	917	915	2	3	99.67	99.78
**sel872**	990	988	0	2	99.80	100.00
**sel873**	859	857	0	1	99.88	100.00
**sel883**	892	890	30	36	95.96	96.61
**sel891**	1267	1265	0	1	99.92	100.00
**sel16265**	1031	1029	10	11	98.93	99.03
**sel16272**	851	849	0	1	99.88	100.00
**sel16273**	1112	1110	4	5	99.55	99.64
**sel16420**	1063	1061	0	1	99.91	100.00
**sel16483**	1087	1085	1	2	99.82	99.91
**sel16539**	922	920	0	1	99.89	100.00
**sel16773**	1008	1006	168	328	67.43	80.17
**sel16786**	925	923	0	1	99.89	100.00
**sel16795**	761	759	0	1	99.87	100.00
**sel17453**	1047	1045	0	1	99.90	100.00
**sele0104**	804	802	0	1	99.88	100.00
**sele0106**	897	894	0	1	99.89	100.00
**sele0107**	823	810	25	34	95.81	96.88
**sele0110**	872	870	1	3	99.66	99.88
**sele0111**	908	906	1	1	99.89	99.89
**sele0112**	684	682	121	189	72.33	80.33
**sele0114**	698	696	23	28	95.98	96.68
**sele0116**	560	557	1	3	99.46	99.82
**sele0121**	1434	1432	2	2	99.86	99.86
**sele0122**	1414	1412	0	1	99.93	100.00
**sele0124**	1121	1119	4	5	99.55	99.64
**sele0126**	945	943	83	793	16.00	64.53
**sele0129**	672	670	40	55	91.80	93.90
**sele0133**	840	838	0	1	99.88	100.00
**sele0136**	810	808	3	4	99.51	99.63
**sele0166**	813	811	0	1	99.88	100.00
**sele0170**	897	895	0	1	99.89	100.00
**sele0203**	1246	1244	0	4	99.68	100.00
**sele0210**	1063	1061	0	1	99.91	100.00
**sele0211**	1575	1573	0	1	99.94	100.00
**sele0303**	1045	1043	1	2	99.81	99.90
**sele0405**	1216	1214	0	57	95.30	100.00
**sele0406**	959	957	0	1	99.90	100.00
**sele0409**	1737	1735	0	1	99.94	100.00
**sele0411**	1202	1200	0	2	99.83	100.00
**sele0509**	1028	1026	0	39	96.20	100.00
**sele0603**	869	867	30	84	90.33	96.32
**sele0604**	1031	1029	0	2	99.81	100.00
**sele0606**	1442	1440	0	4	99.72	100.00
**sele0607**	1184	1182	0	0	100.00	100.00
**sele0609**	1127	1125	3	4	99.64	99.73
**sele0612**	751	749	0	1	99.87	100.00
**sele0704**	1094	1092	0	214	80.40	100.00
**sel30**	1018	1014	0	3	99.70	100.00
**sel31**	1087	1084	45	385	64.52	93.96
**sel32**	1196	1194	0	3	99.75	100.00
**sel33**	527	525	0	4	99.24	100.00
**sel34**	897	895	0	0	100.00	100.00
**sel35**	882	880	0	384	56.36	100.00
**sel36**	948	946	135	227	76.03	84.21
**sel37**	682	679	0	511	24.74	100.00
**sel38**	1563	1561	0	0	100.00	100.00
**sel40**	1171	1169	0	9	99.23	100.00
**sel41**	1069	1067	0	24	97.75	100.00
**sel42**	1366	1363	2	24	98.24	99.85
**sel43**	1247	1245	0	63	94.94	100.00
**sel44**	1430	1427	0	46	96.78	100.00
**sel45**	1337	1335	0	57	95.73	100.00
**sel46**	971	968	66	96	90.09	92.97
**sel47**	856	854	0	98	88.52	100.00
**sel48**	886	884	0	88	90.05	100.00
**sel49**	1398	1396	0	4	99.71	100.00
**sel50**	833	831	0	4	99.52	100.00
**sel51**	661	659	0	32	95.14	100.00
**sel51**	749	747	0	29	96.12	100.00
**sel52**	1411	1409	0	1	99.93	100.00
**sel17152**	1628	1626	0	0	100.00	100.00
**sel14046**	1260	1258	0	0	100.00	100.00
**sel14157**	1081	1079	0	9	99.17	100.00
**sel14172**	663	661	0	73	88.96	100.00
**sel15814**	1036	1034	0	34	96.71	100.00

	111201	110696	1016	4840	95.00	98.59

**Table 4:** T waves detection performance comparison on the QT database [24]. (N/R: not reported).

**Publication**	**Method**	**# Beats**	**Annotation (File Name)**	**SE**	**+P**
This work	Blocks of interest	111,201	Automatic (.pu)	95.0	98.59
This work	Blocks of interest	3542	Manual (.q1c)	98.90	98.77
Martinez *et al.* [38]	Wavelet	3542	Manual (.q1c)	99.77	97.79
Laguna *et al.* [39]	Low-pass-differentiator	3542	Manual (.q1c)	99.0	97.74
Vila *et al.* [40]	Modelling	3542	Manual (.q1c)	96.2	N/R
